# miR-657 Promotes Macrophage Polarization toward M1 by Targeting FAM46C in Gestational Diabetes Mellitus

**DOI:** 10.1155/2019/4851214

**Published:** 2019-12-13

**Authors:** Pingping Wang, Zengfang Wang, Guojie Liu, Chengwen Jin, Quan Zhang, Shuhong Man, Zengyan Wang

**Affiliations:** ^1^Department of Gynecology and Obstetrics, Weifang Hospital of Maternal and Child Health, Weifang 261000, China; ^2^Functional Laboratory, Clinical Medicine College of Weifang Medical University, Weifang 261000, China; ^3^Department of Cardiology, Clinical Medicine College, Weifang Medical University, Weifang 261000, China; ^4^Department of Gynecology, People's Hospital of Weifang, Weifang 261000, China; ^5^Operating Room, Zhucheng People's Hospital, Zhucheng 262200, China

## Abstract

MicroRNA (miRNA) has been widely suggested to play a vital role of in the pathogenesis of gestational diabetes mellitus (GDM). We have previously demonstrated that miR-657 can regulate macrophage inflammatory response in GDM. However, the role of miR-657 on M1/M2 macrophage polarization in GDM pathogenesis is not clear yet. This study is aimed at elucidating this issue and identifying novel potential GDM therapeutic targets based on miRNA network. miR-657 is found to be upregulated in placental macrophages demonstrated by real-time PCR, which can enhance macrophage proliferation and migration in vitro. Luciferase reporter assay shows the evidence that FAM46C is a target of miR-657. In addition, miR-657 can promote macrophage polarization toward the M1 phenotype by downregulating FAM46C in macrophages. The present study strongly suggests miR-657 is involved in GDM pathogenesis by regulating macrophage proliferation, migration, and polarization via targeting FAM46C. miR-657/FAM46C may serve as promising targets for GDM diagnosis and treatment.

## 1. Introduction

Gestational diabetes mellitus (GDM) is one of the most common complications during pregnancy, which causes more and more burden to public health due to its increasing incidence [1]. Both the GDM pregnant women and the infants are at an elevated risk of complications, such as gestational hypertension and preeclampsia for mothers and hyperbilirubinemia, hypocalcemia, and respiratory distress syndrome for babies [2, 3]. Therefore, early screening and management of GDM is essential [4]. Available data has demonstrated the pivotal role of genetics and environmental factors in the development of GDM, but its precise pathogenesis is not yet clear. Insulin resistance and disruption of glucose and insulin balance during pregnancy usually causes GDM. Besides increased levels of estrogen, progesterone, and cortisol during pregnancy, dysregulated placental immunity attributed to various inflammatory cells and their generated inflammation-related mediators in placenta can also induce insulin resistance and thus lead to GDM, such as placental macrophages, dendritic cells, and Th1 cells [5, 6]. The diagnosis of GDM is often missed due to its complicated pathogenesis and lack of reliable biological markers for GDM screening and monitoring during pregnancy.

MicroRNAs (miRNAs) are small noncoding RNAs, which are considered as key regulators of gene expression at the posttranscriptional level and multiple pathophysiological processes [7, 8]. Accumulated studies have strongly suggested miRNAs are essential in regulating pancreatic *β* cell functions, the release of insulin, and insulin resistance [9]. A number of miRNAs have been identified as promising biomarkers for the diagnosis of GDM, including miR-16-5p, miR-375, and the let-7 family [10, 11]. miR-657 is a newly identified regulator involved in inflammation and immunity, which is reported to be associated with type 2 diabetes by controlling insulin growth factor 2 receptor (IGF2R) in a polymorphic manner [12]. We have previously found miR-657 is dysregulated in placenta and participates in GDM by regulating inflammatory response [13]. However, the role of miR-657 on macrophage-mediated immunity and inflammation regulations in GDM still remains vague. The present study is aimed at elucidating this subject by a series of experiments in vitro and providing an updated insight on the GDM pathogenesis.

## 2. Material and Methods

### 2.1. Patients

GDM (*N* = 30) and normal (*N* = 29) pregnancies are enrolled in the current study. All GDM patients terminate pregnancy via elective cesarean section. GDM patients are included strictly based on the criteria, and those with complications, such as hypertension and hyperglycemia, are all excluded.  Table 1 lists the summarized characteristics of patents and controls. Patients and controls have approved and signed the informed consent. The hospital's Institutional Ethics Committee of Weifang Hospital of Maternal and Child Health approves and supervises the present study.

### 2.2. Cells and Tissues

The placental tissues are cut and divided into small pieces immediately after delivery, which were frozen in liquid nitrogen or freshly used for placental mononuclear cells isolation. Phosphate buffer solution (PBS) is applied to wash placental tissues for several times, and the extracted cells are filtrated to remove excess tissues. Placental mononuclear macrophages are isolated by density gradient centrifugation. CD14-positive microbeads (Miltenyi Biotec, San Diego, CA) are used for isolating the placental macrophages according to the protocols. The THP-1 cell line is cultured in RPMI 1640 plus 10% fetal bovine serum (Gibco, USA) and induced into macrophages by the use of 100 nM phorbol-12-myristate-13 acetate (Sigma, USA) under stimulation for 48 hours. THP-1 macrophages are differentiated into M1-like cells stimulated by LPS and IFN-*γ* (Sigma-Aldrich, USA) and M2-like cells by IL-4 (Sigma-Aldrich, USA) in vitro. miR-657 mimics, miR-657 mimic negative control, miR-657 inhibitors, and miR-657 inhibitor negative control, are constructed by the GeneChem Company (Shanghai, China). We apply lentivirus plasmids to make miR-657 and family with sequence similarity 46 member C (FAM46C) overexpressed in THP-1 macrophages.

### 2.3. Cell Proliferation Assay

We use Vazyme Biotech cell counting kit (CCK-8, Nanjing, China) to evaluate the proliferation of THP-1 macrophages in this study. 100 *μ*L/well THP-1 macrophages (5 × 10^5^/mL) are seeded into a 96-well plate overnight. Cells are treated by the miR-657 mimics miR-657 mimic negative control, miR-657 inhibitors, and miR-657 inhibitor negative control for 24, 48, and 72 hours, respectively. Then, a 10 *μ*L CCK-8 solution is added into each well. After incubation for 2 hours, the optical density (OD) is determined at 450 nm by the use of a microplate reader. Experiments are repeated for three independent times.

### 2.4. Transwell Cell Migration Assay

1 × 10^6^/mL THP-1 macrophages are incubated in the incubator overnight. After being transfected by the miR-657 mimics miR-657 mimic negative control, miR-657 inhibitors, and miR-657 inhibitor negative control for 48 hours, the cells are digested, washed, resuspended, and seeded in the transwell chamber for another 24 hours. Cells are fixed by 4% paraformaldehyde for 30 minutes at room temperature. Finally, the migration status of macrophages is estimated under a microscope. We perform three independent determinations.

### 2.5. Quantitative Real-Time Polymerase Chain Reaction (PCR)

Total RNAs are extracted placental tissue macrophages using TRIzol reagent (Invitrogen, USA) according to the instructions. The concentration and purity are estimated by an ultraviolet spectrophotometer at 260 nm and 280 nm. The value of OD is calculated. In total, 0.5 *μ*g RNAs are used for cDNA synthesis by the TAKARA PrimeScript™ RT reagent kit and PCR by TAKARA SYBR Premix (Tianjin, China). The TaqMan miRNA real-time PCR assay kit (ThermoFisher Scientific, USA) is adopted to analyze the expression of miR-657 in primary macrophages and THP-1 cells. Primers for human genes are as follows: TNF-*α*, forward primer, ATGTGGCAAGAGATGGGGAA; reverse primer, CTCACACCCCACATCTGTCT. IL-12, forward primer, TCAGAATTCGGGCAGTGACT; reverse primer, AGTCCCATCCTTCTTTCCCC. Arginase 1, forward primer, ACGGAAGAATCAGCCTGGTG; reverse primer, GTCCACGTCTCTCAAGCCAA. CD206, forward primer, TGGGAGTGCCATCAAAAACG; reverse primer, CCCGATCCCTTGTAGAGCAT. GAPDH, forward primer, ACCACAGTCCATGCCATCAC; reverse primer, TCCACCACCCTGTTGCTGTA.

### 2.6. Western Blot

1 mL/well Solarbio RIPA lysis buffer (Beijing, China) is used to lyse macrophages after transfection by the miR-657 mimics and the corresponding negative control. The extracted proteins are denaturalized in boiling water for 10 minutes. The concentration of proteins is determined by the Bradford BCA protein assay kit (Beyotime, Shanghai, China) based on the protocol. Specific antibodies of CD206 (R&D Systems, USA), Arginase 1 specific antibody (R&D Systems, USA), FAM46C (Abcam, Germany), and GAPDH (Cell Signaling Technology, USA) are adopted to detect their expression in macrophages.

### 2.7. Enzyme-Linked Immune Sorbent Assay (ELISA)

As previously described [13], THP-1 macrophages are incubated overnight and then transfected by lentivirus plasmids or miR-657 mimics and the negative controls for 48 hours. Then, IL-12 and TNF-*α* in supernatant are detected by ELISA based on the protocol of ELISA kits (R&D Systems, USA). Experiments are conducted for three times to detect the OD value at an appropriate wavelength for each factor.

### 2.8. Luciferase Reporter Assay

We construct the wild-type (WT) and mutant (MUT) type of the 3′ untranscriptional region (3′UTR) of FAM46C to the downstream of the firefly luciferase reporter gene of pGL3 vectors. The activity of the luciferase is detected using the dual luciferase reporter assay system (Promega, Madison, USA). Experiments are performed for three times.

### 2.9. Immunofluorescence

THP-1 macrophages (1 × 10^6^/mL) are seeded into a 6-well plate overnight. Then, cells are treated by lentivirus plasmids or miR-657 mimics or controls for 48 hours. The expression of Arginase 1 in macrophages is detected by immunofluorescence by the use of the PE-conjugated Arginase 1 specific antibody (R&D Systems, USA). The product instruction is followed for detection. Experiments are done three times.

### 2.10. Statistical Analysis

All data are shown by mean ± SEM. Prism 6 software (GraphPad Software Inc., USA) is used in our study. An independent sample *T* test or a single-factor variance analysis is applied for statistical analysis. The two-tailed *P* < 0.05 is regarded as significant between groups.

## 3. Results

### 3.1. miR-657 Is Positively Associated with M1 Phenotype Markers of Placental Macrophages

**Figure 1:**
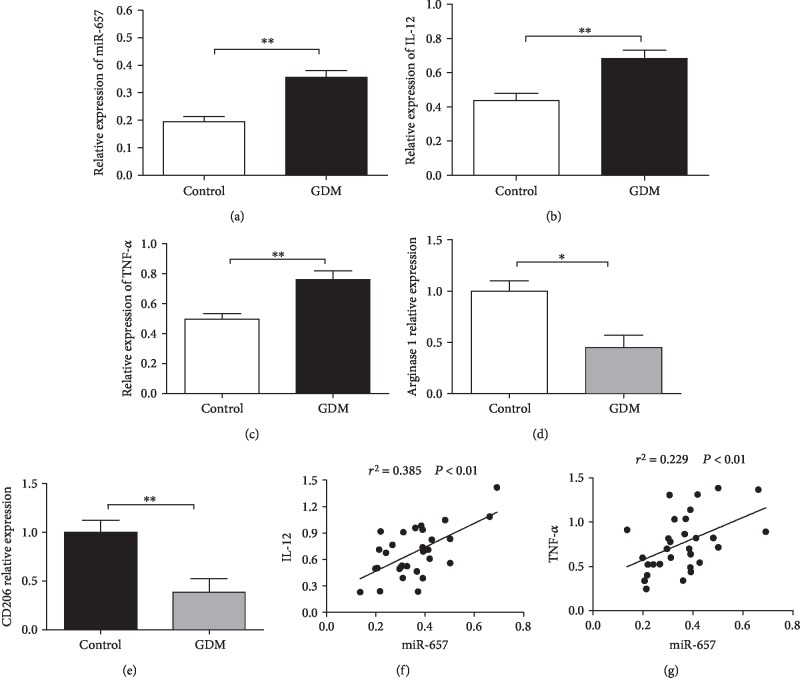
miR-657 is increased and positively associated with M1 markers of placental macrophages in GDM. (a) Increased expression of miR-657 in GDM placental macrophages compared with controls (^∗∗^*P* < 0.01; *N*/*N*, 30/29). (b) mRNA level of IL-12 in GDM placental macrophages in contrast to normal controls (^∗∗^*P* < 0.01; *N*/*N*, 30/29). (c) mRNA level of TNF-*α* in GDM placental macrophages compared with normal pregnancies (^∗∗^*P* < 0.01; *N*/*N*, 30/29). (d) mRNA level of Arginase 1 in placental macrophages of GDM pregnancies in contrast to controls (^∗^*P* < 0.05; *N*/*N*, 30/29). (e) mRNA level of CD206 in placental macrophages of GDM pregnancies compared with controls (^∗∗^*P* < 0.01; *N*/*N*, 30/29). (f) Positive association between miR-657 and IL-12 in GDM placental macrophages (*N* = 30). (g) Positive association between miR-657 and TNF-*α* in GDM placental macrophages (*N* = 30).

**Figure 2:**
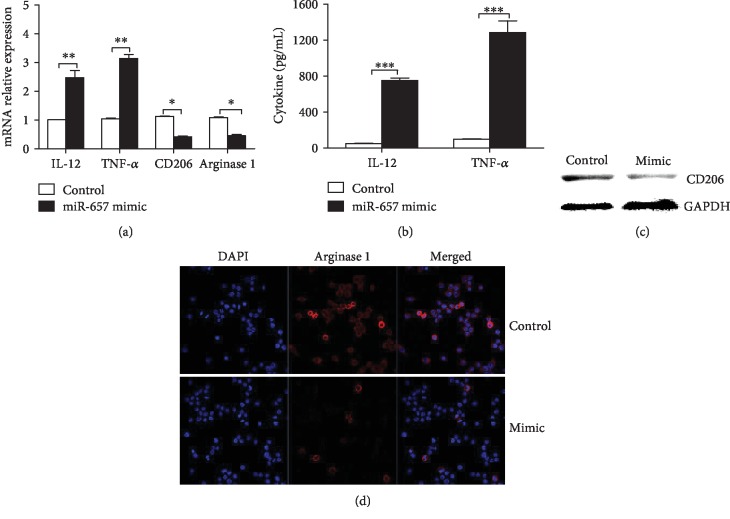
miR-657 is associated with the polarization of macrophages in GDM. (a) mRNA expression of IL-12, TNF-*α*, CD206, and Arginase 1 in miR-657 mimics treated macrophages (^∗^*P* < 0.05; ^∗∗^*P* < 0.01; representative data of three independent experiments). (b) Expression of IL-12 and TNF-*α* in the supernatant of miR-657 mimics treated THP-1 cells (^∗∗∗^*P* < 0.001; data of three repeated experiments). (c) Expression of CD206 protein in THP-1 cells determined by western blot (representative picture of three independent tests). (d) Arginase 1 is decreased in miR-657 mimics treated THP-1 cells as evidenced by immunofluorescence (representative picture of three independent detections).

Elevated miR-657 expression is observed in placental macrophages from GDM patients compared with that in controls (Figure 1(a)). The mRNA level of M1 subtype markers (IL-12 and TNF-*α*) is significantly elevated, whereas the mRNA level of M2 subtype markers (Arginase 1 and CD206) is significantly reduced in placental macrophages of GDM pregnancies when compared with the normal controls (Figures 1(b)–1(e)). More interestingly, there is positive association between miR-657 and IL-12 and TNF-*α* regarding their mRNA expression in GDM placental macrophages (Figures 1(f) and 1(g)). THP-1 macrophages are differentiated into M1-like cells under the stimulation of LPS and IFN-*γ* and induced into M2-like cells by IL-4 in vitro. The expression of IL-12 and TNF-*α* is increased in miR-657 mimics treated THP-1 macrophages (Figures 2(a) and 2(b)), while the expression of CD206 and Arginase 1 is obviously decreased in miR-657 mimics treated macrophages (Figures 2(a), 2(c), and 2(d)). Accordingly, we hypothesize that miR-657 may be involved in placental immunity by regulating the differentiation and polarization of macrophages in GDM.

### 3.2. miR-657 Enhances Macrophage Proliferation and Migration

**Figure 3:**
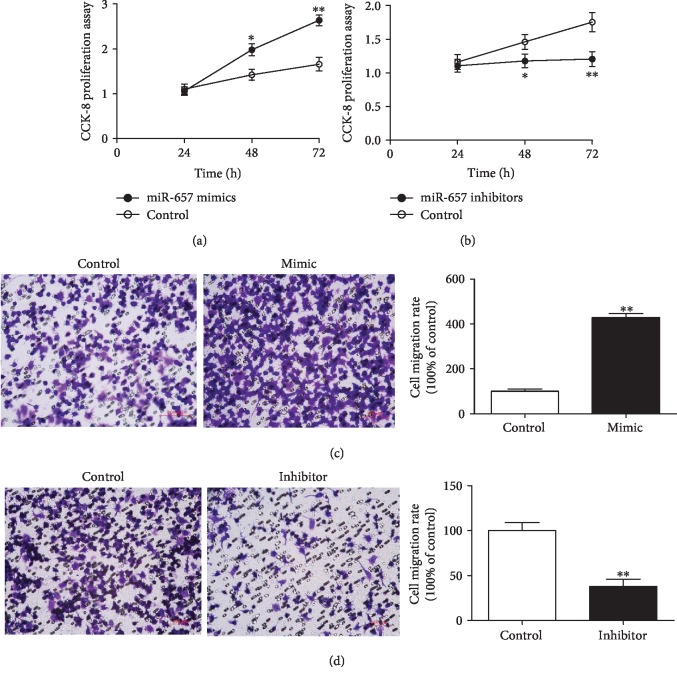
miR-657 regulates macrophage proliferation and migration in vitro. (a) miR-657 mimics increase the proliferation of THP-1 cells in a time-dependent manner (^∗^*P* < 0.05; ^∗∗^*P* < 0.01; data of three independent analyses). (b) miR-657 inhibitors inhibit THP-1 cell proliferation in a time-dependent manner (^∗^*P* < 0.05; ^∗∗^*P* < 0.01; three independent tests). (c) miR-657 mimics promote macrophage migration (^∗∗^*P* < 0.01; representative data of three independent tests). (d) miR-657 inhibitors decrease macrophage migration (^∗∗^*P* < 0.01; representative picture of three independent experiments).

As shown in Figures 3(a) and 3(b), miR-657 mimics enhance the proliferation of THP-1 macrophages, while miR-657 inhibitors inhibit macrophage proliferation determined at different time points (48 h and 72 h). Besides, miR-657 mimics promote the migration of THP-1 macrophages, whereas downregulation of miR-657 in THP-1 macrophages can inhibit their migration (Figures 3(c) and 3(d)). Taken together, miR-657 is capable of enhancing macrophage proliferation and migration in vitro.

### 3.3. FAM46C Is a Target of miR-657

**Figure 4:**
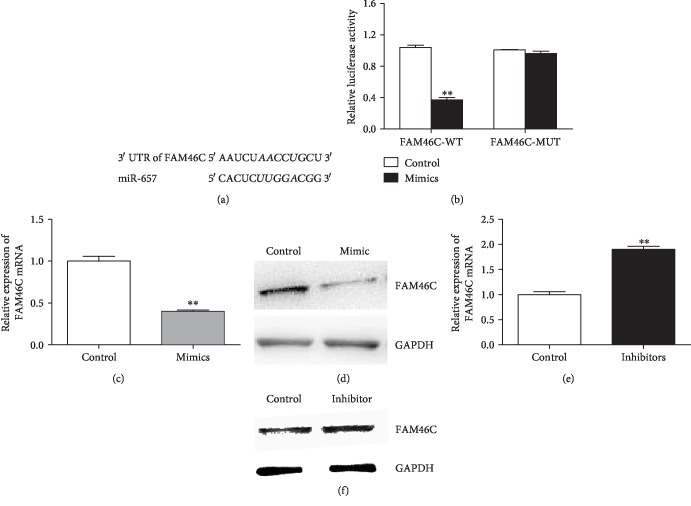
miR-657 can targetly regulate FAM46C. (a) miR-657 specially recognizes the 3′UTR of FAM46C. (b) Luciferase reporter assay shows FAM46C is the direct target of miR-657 (^∗∗^*P* < 0.01; representative data of three repeated tests). (c) Decreased mRNA expression of FAM46C in miR-657 mimics treated THP-1 macrophages (^∗∗^*P* < 0.01; representative data of three repeated determinations). (d) Decreased protein expression of FAM46C in miR-657 mimics treated THP-1 macrophages determined by western blot (representative picture of three repeated tests). (e) Elevated mRNA expression of FAM46C in miR-657 inhibitors treated THP-1 cells (^∗∗^*P* < 0.01; *n* = 3). (f) Increased protein expression of FAM46C in miR-657 inhibitors treated macrophages (representative picture of three independent experiments).

To investigate the mechanism of miR-657 in regulating macrophages, we perform bioinformatics analysis to screen the potential targeted genes of miR-657. After searching in the TargetScan database, we find that miR-657 can specially recognize the 3′UTR of FAM46C (Figure 4(a)). Luciferase reporter assay shows evidence that FAM46C is a target of miR-657 (Figure 4(b)). Besides, the expression of FAM46C is obviously decreased when THP-1 macrophages are treated by miR-657 mimics (Figures 4(c) and 4(d)). However, miR-657 inhibitors can enhance the expression of FAM46C (Figures 4(e) and 4(f)). Accordingly, we hypothesize that miR-657 can regulate functions of macrophage by targeting FAM46C.

### 3.4. miR-657 Promotes Macrophage Polarization toward M1 by Targeting FAM46C

**Figure 5:**
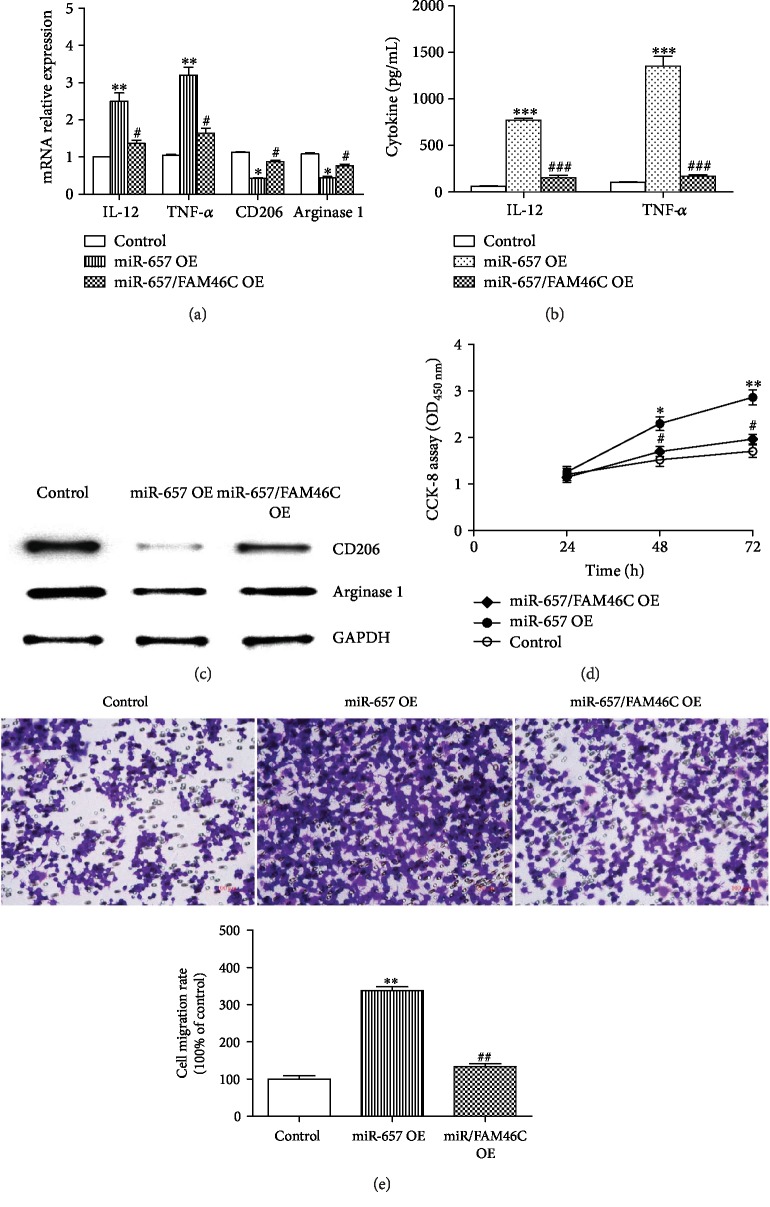
miR-657 promotes macrophage polarization toward M1 via FAM46C. (a) mRNA expression of IL-12, TNF-*α*, Arginase 1, and CD206 in miR-657/FAM46C overexpressed THP-1 macrophages (compared with the control group, ^∗^*P* < 0.05, ^∗∗^*P* < 0.01; compared with the miR-657 overexpressed (OE) group, ^#^*P* < 0.05; *n* = 3). (b) Expression of IL-12 and TNF-*α* in the cultural supernatant of THP-1 cells (compared with the control group, ^∗∗∗^*P* < 0.001; compared with the miR-657 OE group, ^###^*P* < 0.001; *n* = 3). (c) Western blot estimating the protein expression of Arginase 1 and CD206 in macrophages (representative picture of three repeated tests). (d) The proliferation status of miR-657/FAM46C overexpressed macrophages (compared with the control group, ^∗^*P* < 0.05, ^∗∗^*P* < 0.01; compared with the miR-657 OE group, ^#^*P* < 0.05; *n* = 3). (e) Transwell assay estimating the migration of miR-657/FAM46C overexpressed THP-1 cells (compared with the control group, ^∗∗^*P* < 0.01; compared with the miR-657 OE group, ^##^*P* < 0.01; *n* = 3).

For further elucidation, we perform gene compensation experiments by overexpression of miR-657 and FAM46C simultaneously in THP-1 macrophages. As seen in Figures 5(a)–5(c), obviously elevated expression of M1 markers (IL-12 and TNF-*α*) but reduced M2 markers (Arginase 1 and CD206) is found in the miR-657 overexpressed THP-1 macrophages. However, FAM46C rescues the effect of miR-657 when it is co-overexpressed in macrophages (Figures 5(a)–5(c)). In addition, macrophage proliferation and migration are also inhibited when miR-657 and FAM46C are simultaneously overexpressed in THP-1 macrophages, whereas miR-657 overexpression can promote the proliferation and migration of macrophages (Figures 5(d) and 5(e)). Taken together, miR-657 promotes macrophage polarization toward M1 by targeting FAM46C, while upregulation of FAM46C can rescue the effect of miR-657 on macrophage growth and polarization.

## 4. Discussion

GDM is defined as any degree of carbohydrate intolerance during pregnancy, the prevalence of which is rising worldwide. This study firstly reports that miR-657 participates in GDM pathogenesis by promoting macrophage proliferation, migration, and polarization toward M1 by downregulating FAM46C in GDM. It provides new insight into GDM pathogenesis and demonstrates promising biomarkers for GDM diagnosis and treatment.

Accumulating studies have suggested miRNAs are closely related to glycometabolic disorders including GDM by regulating placental immunity, pancreatic *β* cell functions, insulin sensitivity, and insulin resistance [14–16]. Circulating miRNAs contributing to tissue cross-talk have also been identified as potential biomarkers for GDM [17]. As a result, miRNAs are crucial for the development of GDM, but how specific miRNAs regulate placental immunity and glucose metabolism remains not much clear. Latreille et al. have found that miR-7a leads to *β* cell dedifferentiation and impaired insulin secretion in transgenic mice [18]. Reversely, the study by Chen et al. shows the evidence that miR-351 protects against insulin resistance and liver gluconeogenesis via targeting flotillin 2 (FLOT2) in GDM mice, supporting a pivotal role for miR-351 in maintaining the glycometabolism balance [19]. It has also been well documented that certain miRNAs are involved in GDM pathogenesis by influencing inflammatory conditions, monocyte chemotaxis, and angiogenesis [20, 21]. Previously, we have found that dysregulation of miR-657 affects placental inflammatory response in GDM via regulating the IL-37/NF-*κ*B signaling axis [13]. Results in this study reveal that miR-657 is increased in placental macrophages of GDM pregnancies compared with that in normal pregnancies. Besides, it enhances macrophage proliferation and migration when overexpressing in macrophages. Moreover, miR-657 is positively associated with inflammatory cytokines of IL-12 and TNF-*α* regarding their expression in placental macrophages. IL-12 and TNF-*α* are key markers for M1 subtype macrophages. Accordingly, miR-657 may be involved in placental immunity by regulating macrophage proliferation, migration, and functions. However, how miR-657 regulates macrophage polarization and functions is still unknown.

The status of macrophage polarization in placenta can affect maternal tolerance and pregnancy outcomes [22–24]. M2 macrophages possess anti-inflammatory and tissue remodeling functions. The M2 phenotype is essential for maintaining the maternal-fetal interface immune tolerance and protecting the fetus from inflammatory microenvironments in the placenta [22]. Many studies have implicated that miRNAs confer important effects on macrophage polarization in kinds of diseases [25–27]. Accumulating studies have well documented that some miRNAs are capable of regulating inflammation and insulin resistance in diabetes by influencing macrophage polarization [28–30]. Nonetheless, little is known of the altering effects of miRNAs on GDM development and progression by regulating macrophage differentiation and functions. The study by Schliefsteiner et al. has reported that an anti-inflammatory M2 phenotype of human placental Hofbauer cells from 6 GDM patients was documented compared with cells from 5 healthy women [22]. However, the M1 marker CD86 has also been found to be increased, although only by trend [22]. In our study, an M1 proinflammatory phenotype of placental macrophages has been found in GDM pregnancies compared with healthy controls. Causes for the difference may be attributed to a different sample size, statistical power, ethnicity of participants, diagnosis in diverse trimesters, and so on. In addition, we, for the first time, demonstrate that miR-657 can promote macrophages polarized toward M1 in GDM. To further explore the effect of miR-657 on macrophage functions, we perform bioinformatics analysis and find that miR-657 is capable of recognizing the 3′UTR of FAM46C. Subsequently, FAM46C is demonstrated to be the targeted gene of miR-657 by luciferase reporter assay. Furthermore, findings in this study have strongly suggested that miR-657 facilitates macrophages to polarize towards M1-like cells via targeting FAM46C in macrophages. Some studies have supported the essential role of miRNAs in the regulation of pancreatic *β* cell functions and the downstream insulin signaling pathway [31, 32]. The phosphoinositide 3-kinase/protein kinase B (PI3K/AKT) pathway is the classical signaling pathway that regulates islet *β* cell function and insulin sensitivity and resistance mediated by miRNAs in glucose metabolism [19, 31]. Nevertheless, the modifying effects of miR-657 on pancreatic *β* and its downstream molecular mechanism are not well elucidated in the present study. More future studies are warranted for further investigation.

In summary, the current study shows strong evidence that miR-657 participates in GDM by promoting macrophage proliferation, migration, and polarization toward M1. FAM46C is the target of miR-657. miR-657/FAM46C axis may serve as promising targets for GDM diagnosis and treatment. However, the downstream molecule mechanism needs to be investigated in future studies to provide updated insight into the pathogenesis of GDM.

## Figures and Tables

**Table 1:** Characteristics.

	Patients (30)	Control (29)	*P* value
Age (years)	28-40	29-38	*P* > 0.05
Gestational weeks (weeks)	37.9 ± 1.1	39.2 ± 1.1	*P* > 0.05
Mother weight (kg)	70.6 ± 5.5	64.2 ± 7.4	*P* > 0.05
Birth weight of infant (kg)	3.9 ± 1.1	3.2 ± 1.2	*P* > 0.05
Blood pressure			
SBP (mmHg)	119.4 ± 5.3	114.4 ± 4.2	*P* > 0.05
DBP (mmHg)	69.9 ± 4.7	68.2 ± 4.9	*P* > 0.05
Glucose metabolism index			
Fasting insulin (mIU/L)	10.8 ± 1.6	7.7 ± 1.2	*P* < 0.01
Fasting glucose (mmol/L)	4.9 ± 0.5	3.9 ± 0.4	*P* < 0.01
1 h glucose (mmol/L)	9.2 ± 1.6	5.8 ± 1.7	*P* < 0.01
2 h glucose (mmol/L)	8.8 ± 1.4	5.1 ± 1.1	*P* < 0.01

## Data Availability

The data used to support the findings of this study are available from the corresponding author upon request.
